# The preliminary study suggests an association between NF-ĸB pathway activation and increased plasma 20S proteasome activity in intracranial aneurysm patients

**DOI:** 10.1038/s41598-024-54692-8

**Published:** 2024-02-16

**Authors:** Joanna Kamińska, Marzena Tylicka, Kinga Sutkowska, Karolina Marta Gacuta, Magdalena Maria Sawicka, Ewa Kowalewska, Magdalena Ćwiklińska-Dworakowska, Mateusz Maciejczyk, Tomasz Łysoń, Johannes Kornhuber, Piotr Lewczuk, Joanna Matowicka-Karna, Olga Martyna Koper-Lenkiewicz

**Affiliations:** 1 Department of Clinical Laboratory Diagnostics, Medical University of Bialystok, 15A Jerzego Waszyngtona St., 15-269 Białystok, Poland; 2 Department of Biophysics, Medical University of Bialystok, 2A Adama Mickiewicza St., 15-089 Białystok, Poland; 3 Department of Clinical Laboratory Diagnostics, Clinical Hospital of the Medical University of Bialystok, 15A Jerzego Waszyngtona St., 15-269 Białystok, Poland; 4 Department of Analysis and Bioanalysis of Medicines, Medical University of Bialystok, 2D Mickiewicza St., 15-222 Białystok, Poland; 5 Department of Oncological Surgery and General Surgery, Independent Public Health Care Facility of the Ministry of the Interior and Administration in Bialystok named Marian Zyndram-Kościałkowski, 27 Fabryczna St., 15-471 Białystok, Poland; 6 Department of Hygiene, Epidemiology, and Ergonomics, Medical University of Białystok, 2C Mickiewicza St., 15-022 Białystok, Poland; 7 Department of Neurosurgery, Medical University of Bialystok/Clinical Hospital of the Medical University of Bialystok, 24A Marii Skłodowskiej-Curie St., 15-276 Białystok, Poland; 8 Department of Psychiatry and Psychotherapy, University Hospital Erlangen, and Friedrich-Alexander-Universität Erlangen-Nürnberg, Erlangen, Germany; 9 Department of Neurodegeneration Diagnostics, Medical University of Bialystok, 15A Jerzego Waszyngtona St., 15-269 Białystok, Poland

**Keywords:** Intracranial aneurysms (IA), 20S proteasome, Canonical NF-κB pathway, NF-κB1, NF-κB2, NF-κB p65, Neuroscience, Biomarkers, Medical research

## Abstract

The significant role of increased activation of 20S proteasomes in the development of abdominal aortic aneurysms has been well-established in a mouse model. The available literature lacks similar studies concerning brain aneurysms. The aim of the study was to verify the hypothesis that patients with unruptured intracranial aneurysms (UIA) have increased 20S proteasome ChT-L activity compared to the control group of individuals without vascular lesions in the brain. In the next step, the relationship between the activity of 20S proteasomes ChT-L and precursor proteins from the nuclear factor kappa-light-chain-enhancer of activated B cells (NF-κB) family, namely NF-κB1 (p105), NF-κB2 (p100), NF-κB p65, and the inflammatory chemokine MCP-1, was examined. Patients with UIA had significantly higher 20S ChT-L proteasome activity compared to the control group. Patients with multiple aneurysms had significantly higher 20S proteasome ChT-L activity compared to those with single aneurysms. In patients with UIA, the activity of the 20S proteasome ChT-L negatively correlated with the concentration of NF-κB1 (p105) and NF-κB p65 precursor proteins and positively correlated with the concentration of the cerebrospinal fluid chemokine MCP-1. Our results may suggest that increased 20S proteasome ChT-L activity in UIA patients modulates inflammation in the cerebral arterial vessel via the MCP-1 chemokine as a result of activation of the canonical NF-κB pathway.

## Introduction

Proteasomes are large macromolecular enzymatic complexes found in the cells of all eukaryotic organisms and in some bacteria^1–3^. The role of proteasomes is the degradation of unnecessary and/or damaged, improperly folded proteins^1^. One of the major form of the proteasome is the 26S proteasome, which is composed of two main subcomplexes: the 20S proteasome, known as the core particle (CP), and the 19S proteasome, known as the regulatory particle (RP, activator PA700)^4^. The 20S proteasome is a cylindrical structure consisting of 4 rings. The catalytic sites are located inside this cylinder, where protein substrates are bound. Enzymatically active are the beta 1 subunit (caspase-like activity), beta 2 subunit (trypsin-like activity), and beta 5 subunit (chymotrypsin-like activity, ChT-L). The 20S proteasome degrades proteins into smaller peptides by the proteolytic route. The 19S proteasome has the ability to binding to ubiquitin-tagged proteins, unfolding them, and feeding them into the 20S proteasome for degradation^5–7^. The 20S proteasome also functions in a self-contained manner, without the need for ubiquitin tagging to facilitate protein degradation^8^. Wada et al.^9^, were the first to demonstrate the presence of the 20S proteasome in human serum, which is referred to as the "circulating proteasome" (c-proteasome). As demonstrated by electron microscopy, purified c-proteasomes are intact particles of the 20S proteasome^10^.

Proteasomes activate the nuclear factor kappa-light-chain-enhancer of activated B cells (NF-κB) transcription factor, which plays a significant role in regulating the expression of approximately 400 genes, including cytokines, chemokines, enzymes, adhesion molecules, cell cycle regulators, and angiogenic molecules^11^. Under physiological conditions, NF-κB is bound to inhibitor κB (IκB). The role of IκB is to keep NF-κB in an inactive form. It has been demonstrated that increased proteasome activity can lead to intensified degradation of IκB or IκB-like proteins, resulting in the activation of NF-κB^12–14^. As a result of activation, homo- or heterodimers of NF-κB are formed, which are translocated to the cell nucleus and influence, in either the canonical or non-canonical pathway, the transcription of genes^14^. NF-κB regulates, among others, the inflammatory response and the migration of inflammatory cells, participating in both innate and acquired immune responses, the maturation of immune cells, the development of secondary lymphoid organs, osteoclastogenesis, as well as in cancer and apoptosis^15–18^.

Previous experimental studies indicate increased expression of NF-κB in a cerebral aneurysm model^19,20^. Additionally, they demonstrate that blocking the activity of NF-κB inhibits the formation and growth of intracranial aneurysms^18,21,22^. In our previous study we showed that the canonical NF-κB signaling pathway with the involvement of the p65 subunit is activated in unruptured intracranial aneurysm (UIA) patients^23^. We also indicated chemokine monocyte chemoattractant protein-1 (MCP-1) as the protein particularly involved in the formation of brain aneurysms^23^.

The aim of the study was to verify the hypothesis whether patients with UIA have increased 20S proteasome ChT-L activity compared to the control group of individuals without vascular lesions in the brain. Because proteasomes can activate the NF-κB transcription factor, in the next step the relationship between the 20S proteasome ChT-L activity and precursor proteins from the NF-κB family, i.e. NF-κB1 (p105), NF-κB2 (p100), NF-κB p65, and the inflammatory chemokine MCP-1 was analyzed.

### Ethical approval statement and patient consent statement

The study was conducted according to the guidelines of the Declaration of Helsinki and the protocol was approved by the Bioethics Human Research Committee of the Medical University of Bialystok (Permission No. APK.002.9.2021). All subjects gave their informed consent for inclusion before they participated in the study.

## Results

### Plasma 20S proteasome ChT-L activity results

**Figure 1:**
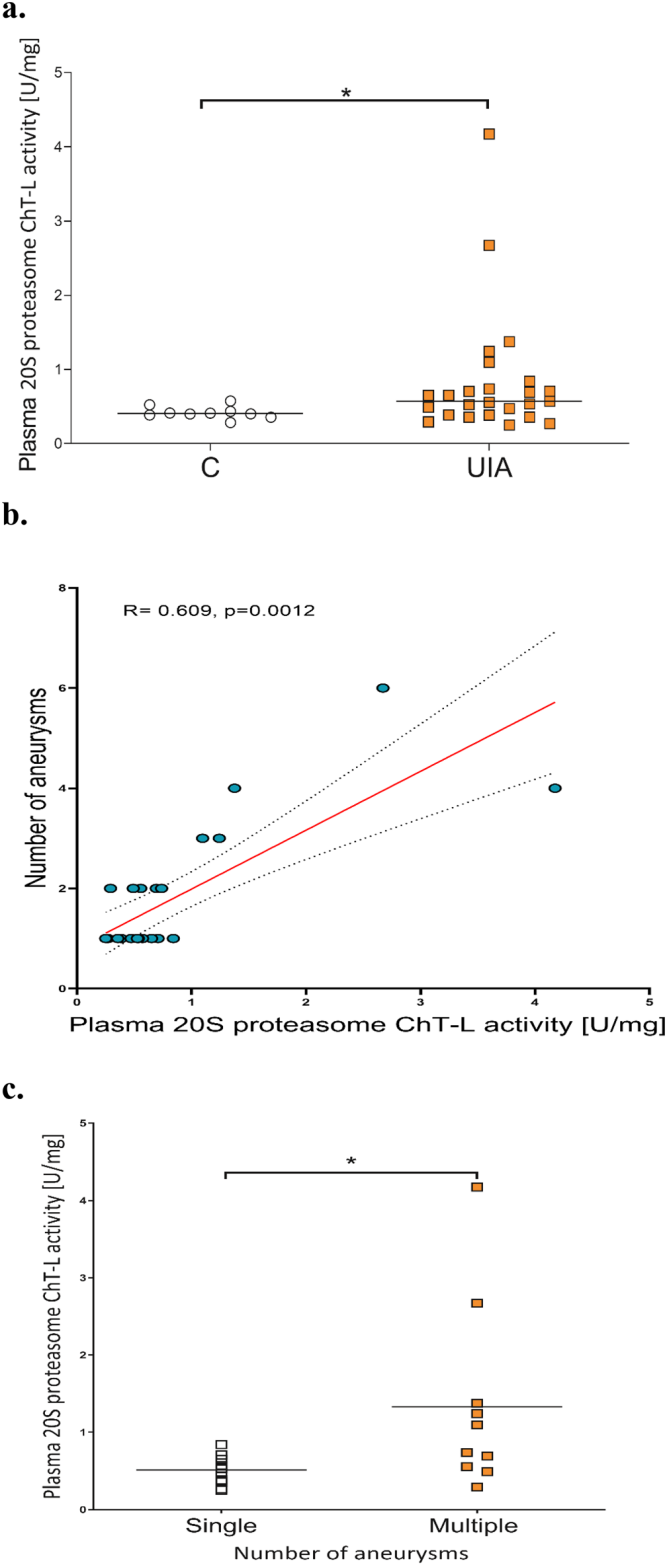
(**a**) Comparison of plasma 20S proteasome ChT-L activity between the unruptured intracranial aneurysm (UIA) group and control individuals without vascular lesions in the brain (C). (**b**) The correlation coefficient analysis between plasma proteasome 20S ChT-L activity and the number of aneurysms. (**c**) Comparison of plasma 20S proteasome ChT-L activity between UIA patients with single aneurysms and those with multiple aneurysms, * p ≤ 0.05.

The median plasma 20S proteasome ChT-L activity in the unruptured intracranial aneurysm (UIA) group was 0.571 U/mg (25th and 75th percentiles: 0.385–0.790 U/mg), which was significantly higher than that in the control group of individuals without vascular lesions in the brain, where it was 0.407 U/mg (25th and 75th percentiles: 0.379–0.457 U/mg) (p = 0.0404) (Fig. 1a). The correlation coefficient analysis indicated that plasma 20S proteasome ChT-L activity positively correlated with the number of aneurysms (R = 0.609, p = 0.0012) (Fig. 1b).

A deeper analysis presented, that UIA patients with multiple aneurysms had significantly higher median plasma 20S proteasome ChT-L activity (0.917 U/mg, 25th and 75th percentiles: 0.540–1.699 U/mg) compared to individuals with single aneurysms (0.527 U/mg, 25th and 75th percentiles: 0.358–0.652 U/mg, p = 0.0137) (Fig. 1c).

**Figure 2:**
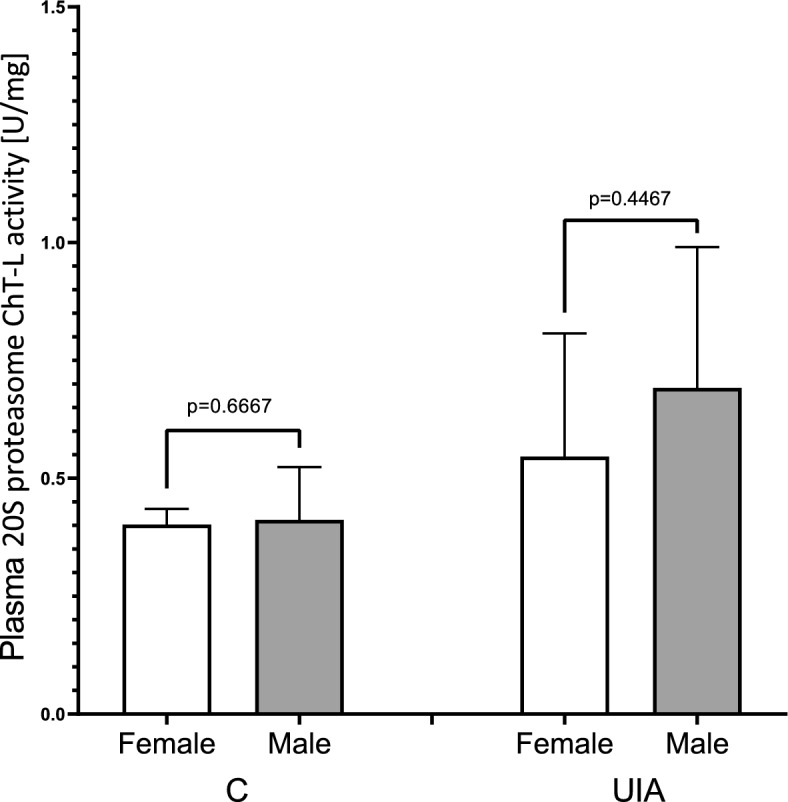
The 20S proteasome ChT-L activity based on gender (female/male) in the UIA and control group. Legend for Fig. 2: C—control group, ChT-L, chymotrypsin-like; UIA, unruptured intracranial aneurysm group.

Considering that the selection of patients for the study and control groups in terms of gender was not ideal (women comprised 80% and 70% of the study group and the control group, respectively), in the next step we evaluated the 20S proteasome ChT-L activity based on gender in both groups. The plasma 20S proteasome ChT-L activity showed no significant differences based on gender (female *vs* male) in both the study and control groups (Fig. 2).

### Serum NF-κB1 (p105) and NF-κB2 (p100) concentration results

**Figure 3:**
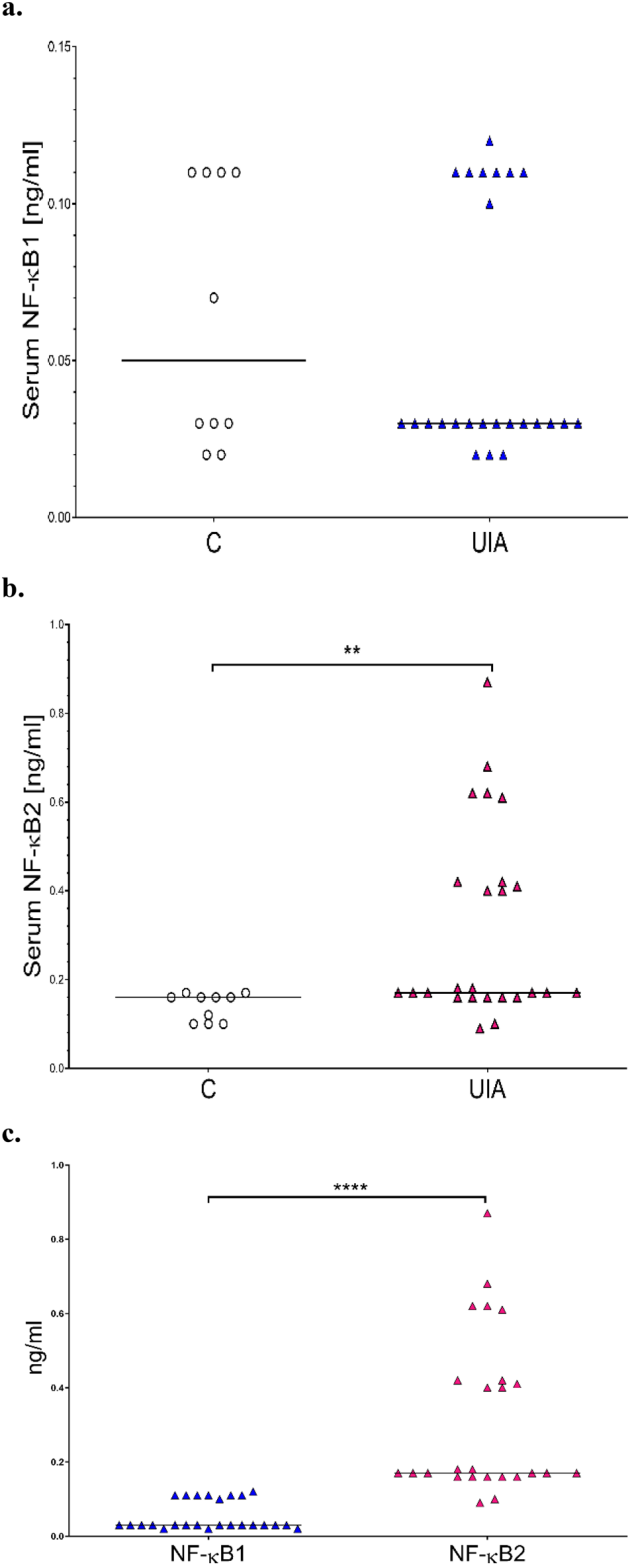
(**a**) Comparison of serum NF-κB1 (p105) concentration between unruptured intracranial aneurysm (UIA) patients and control individuals without vascular lesions in the brain (C). (**b**) Comparison of serum NF-κB2 (p100) concentration between UIA patients and control individuals without vascular lesions in the brain (C). (**c**) Serum NF-κB1 (p105) and NF-κB2 (p100) concentration in UIA patients, **p ≤ 0.01, ****p ≤ 0.0001.

Median serum NF-κB1 (p105) concentration (0.030 ng/ml, 25th and 75th percentiles: 0.030–0.110 ng/ml) in UIA patients was lower (0.050 ng/ml, 25th and 75th percentiles: 0.028–0.110 ng/ml) compared to the control group, but the difference was not significant (p = 0.7568) (Fig. 3a). Median serum NF-κB2 (p100) concentration (0.170 ng/ml, 25th and 75th percentiles: 0.160–0.420 ng/ml) in UIA patients was significantly higher (0.160 ng/ml, 25th and 75th percentiles: 0.100–0.163 ng/ml) compared to the control group (p = 0.0020) (Fig. 3b). Further analysis revealed that in UIA patients, the median serum NF-κB1 (p105) concentration was significantly lower (0.030 ng/ml) compared to the median NF-κB2 (p100) concentration (0.017 ng/ml) (p < 0.0001) (Fig. 3c), however, a significant relationship (p = 0.0022) was also observed in the control group (0.160 *vs* 0.05 ng/ml).

### Correlation coefficient results

**Figure 4:**
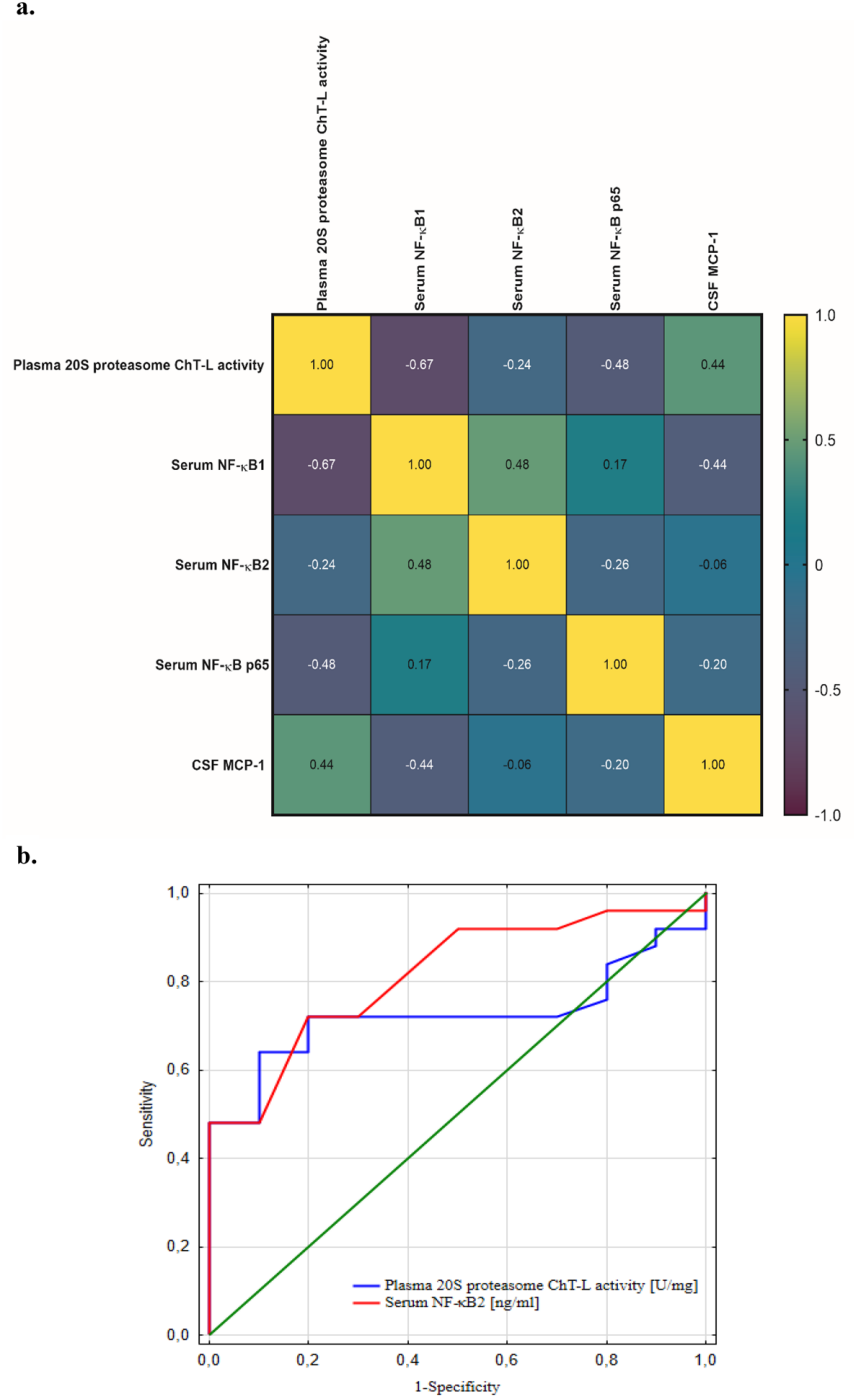
(**a**) The heat map of the complex relationship between plasma 20S proteasome ChT-L activity and serum NF-κB1 (p105), NF-κB2 (p100), NF-κB p65 and cerebrospinal fluid MCP-1 concentrations in UIA patients. The utilized color scale and the R values found in individual fields denote the strength and direction (no minus sign for positive correlation, with a minus sign for negative correlation) of the linear relationship between the two variables. Statistically significant dependencies for the examined parameters have been elucidated in the results section. (**b**) ROC curve for plasma 20S proteasome ChT-L activity and serum NF-κB2 (p100) concentration in differentiating UIA patients from control individuals without vascular lesions in the brain. Legend for Fig. 4a-b: ChT-L, chymotrypsin-like; MCP-1, Monocyte Chemoattractant Protein-1; NF-κB, nuclear factor kappa-light-chain-enhancer of activated B cells; ROC curve, receiver operating characteristic curve; UIA, unruptured intracranial aneurysm.

The heat map presents the complex relationship between plasma 20S proteasome ChT-L activity and the concentrations of serum NF-κB1 (p105), NF-κB2 (p100), NF-κB p65, and cerebrospinal fluid MCP-1 in UIA patients (Fig. 4a). We observed, that plasma proteasome 20S ChT-L activity showed significant, negative correlation with serum NF-κB1 (p105) concentration (R = − 0.67, p = 0.0002) and with serum NF-κB p65 concentration (R = − 0.48, p = 0.0148). Moreover, plasma 20S proteasome ChT-L activity positively correlated with cerebrospinal fluid MCP-1concentration (R = 0.44, p = 0.0290) (Fig. 4a**)**.

### Diagnostic utility analysis

The plasma 20S proteasome ChT-L activity and serum NF-κB2 (p100) concentration were diagnostically useful in differentiating UIA patients from control individuals without vascular lesions in the brain. The AUCs were statistically higher than AUC = 0.5 (p = 0.0081 and p < 0.0001, respectively), which indicates their diagnostic usefulness for differentiating these patients. The AUC for plasma 20S proteasome ChT-L activity equaled 0.724, the diagnostic specificity was 90%, and the positive predictive value equaled 94%. The AUC for serum NF-κB2 (p100) concentration was 0.814, the diagnostic specificity was 80%, and the negative predictive value equaled 90% (Table 1, Fig. 4b).

**Table 1:** Diagnostic parameters in differentiating unruptured intracranial aneurysm patients from control individuals without vascular lesions in the brain.

	Cut-off	Youden index	AUC ± SE	Se [%]	Sp [%]	PPV [%]	NPV [%]	ACC [%]	p-value
20S proteasome ChT-L activity [U/mg]	0.53	0.54	0.724** ± **0.085	64	90	94	50	71	0.0081
NF-κB2 [ng/mL]	0.017	0.52	0.814** ± **0.074	72	80	74	90	53	< 0.0001

ACC, diagnostic accuracy; AUC, area under the ROC curve; ChT-L, chymotrypsin-like; Cut-off (based on the highest Youden index); NPV, negative predictive value; PPV, positive predictive value; Se, diagnostic sensitivity; SE, Standard Error; Sp, diagnostic specificity.

### Power analysis and sample size calculations

The investigating the 20S proteasome ChT-L activity is "trailblazing" in patients with UIA, and as such, we did not conduct a priori (prospective) power analysis and sample size calculations. However, we evaluated the test power based on the values obtained in this study, utilizing an independent two-sided t-test with a 95% confidence level and a margin of error of ± 5%. This analysis yielded a power of 65% (0.65). Additionally, we performed the sample size calculation for a power of 0.8 based on our results, indicating a required sample size of 19 for both the study and control groups.

## Discussion

Our results of 20S proteasome ChT-L activity assessment are the first conducted in patients with unruptured intracranial aneurysms (UIA). We demonstrated statistically significantly higher 20S proteasome ChT-L activity in patients with UIA compared to the control group of individuals without vascular lesions in the brain. In the next stage of the analysis, we correlated the activity of 20S ChT-L proteasomes with the serum concentration of precursor proteins of the NF-κB family—NF-κB1 (p105), NF-κB2 (p100), and NF-κB (p65). In patients with UIA, we observed a statistically significant negative correlation between the activity of 20S ChT-L proteasomes and the concentration of precursor proteins NF-κB1 (p105) and NF-κB (p65). The obtained results may suggest that increased activity of 20S ChT-L proteasomes in patients with UIA modulate the inflammatory state in cerebral arteries through the activation of the canonical NF-κB pathway involving the heterodimer NF-κB p50/p65.

The subunits of NF-κB include NF-κB1 (p50), NF-κB2 (p52), RelA (p65), RelB, and c-Rel^24^. These subunits can combine in various combinations to regulate gene transcription via canonical or non-canonical pathways and play a crucial role in numerous biological processes, including immune response, inflammation, and and cellular differentiation, proliferation, and survival in almost all multicellular organisms^25^. The NF-κB1 (p50) subunit is produced as a precursor protein called p105, which is processed to generate the mature protein. Moorthy et al.^26^ demonstrated that the transformation from the precursor form of NF-κB1 (p105) to the mature form of NF-κB1 (p50) occurs with the involvement of 20S proteasomes, which endoproteolytically (in a ubiquitin-independent manner) cleave fully synthesized precursor molecules of NF-κB1. The mature subunit NF-κB1 (p50) combines with the subunit NF-κB (p65), forming a complex that, through the canonical pathway of NF-κB activity, activates the transcription of genes encoding inflammatory cytokines^26,27^. Studies involving animal models demonstrate the upregulation in the expression of the most common NF-κB p50/p65 heterodimer in both endothelial cells of intracranial aneurysm and macrophages^19,28–30^. In our previous study, we also indicated that the NF‑κB signaling pathway involving the p65 subunit is activated in UIA patients^23^.

In our study, we also observed a statistically significant, positive correlation between 20S proteasome ChT-L activity and the concentration of cerebrospinal fluid chemokine MCP-1. In previous studies, we demonstrated that the chemokine MCP-1 is involved in the formation and development of intracranial aneurysms^23,31^. Aoki et al. emphasize that NF-κB activation may regulate the transcription of MCP-1 chemokine genes and VCAM-1 adhesion molecules, thereby mediating the recruitment of macrophages to the aneurysm wall. In studies conducted on a mouse model, the authors demonstrated that the deficiency or inhibition of the NF-κB (p50) subunit by decoy oligonucleotides or anti-NF-κB agents inhibited the formation and progression of intracranial aneurysms and suppressed the expression and production of pro-inflammatory proteins crucial to their development, such as MCP-1, interleukins, and extracellular matrix metalloproteinases^29,32^. The current results allow us to hypothesize that in patients with UIA, the increased proteasomal transformation of the NF-κB1 protein into its mature form may lead to the abnormal activation of NF-κB p50/p65 heterodimers, which, in turn, induces the transcription of inflammatory cytokine genes, including the MCP-1 chemokine indicated in this study.

In our study, we also showed a significantly higher concentration of the NF-κB2 (p100) subunit in patients with UIA compared to the control group. Furthermore, we noticed that in patients with UIA, the concentration of NF-κB2 (p100) was significantly higher compared to the concentration of NF-κB1 (p105). Based on this, we can assume that NF-κB2 (p100) is sequestered in excess in patients with UIA. It should be emphasized that the altered distribution of NF-κB dimers, including those from the non-canonical pathway, is considered a characteristic feature of chronic inflammatory diseases^27^. The formation of NF-κB2 (p52) is associated with the non-canonical (alternative) pathway of NF-κB activation. Formation of the mature NF-κB2 molecule (p52) may be mediated by proteasomal removal of the C-terminal inhibitory domain from NF-κB2 (p100)^27,33,34^. Furthermore, NF-κB2 (p52), similar to NF-κB1 (p50), can interact with NF-κB (p65), forming a related, smaller heterodimer NF-κB p52/p65, which, like NF-κB p50/p65 through the canonical pathway, regulates the expression of pro-inflammatory genes^35,36^. Thus, non-canonical signaling, represented by the sequestered excess of the NF-κB2 (p100) subunit, may enhance the canonical activity of NF-κB (p65)^23^. It is also worth mentioning that NF-κB1 (p50) can combine with NF-κB2 (p52) to form a high molecular weight NF-κB1 p50/p52 complex that also sequesters the monomeric form of NF-κB (p65) in the cytoplasm^35,37,38^. We demonstrated a statistically significant positive correlation between the NF-κB1 (p105) subunit and NF-κB2 (p100), allowing for the indirect inference that such a complex may be formed in UIA patients, thereby increasing the availability of the NF-κB (p65) form for the canonical pathway.

In our study, we additionally demonstrated a statistically significant positive relationship between the activity of 20S ChT-L proteasomes and the number of aneurysms. In patients with multiple aneurysms, the activity of 20S ChT-L proteasomes was statistically higher compared to those with single aneurysms. The assessment of diagnostic utility showed that the activity of 20S ChT-L proteasomes may be useful in differentiating patients with UIA from individuals without vascular lesions in the brain, further suggesting the involvement of 20S ChT-L proteasomes in the development of brain aneurysms.

In the literature, there are only single studies assessing proteasomal activity in an abdominal aortic aneurysm model^39,40^. The authors emphasize in these studies that the activation of 20S proteasomes plays a crucial role in the development of abdominal aortic aneurysms in a mouse model^39^. Additionally, they indicate that the 20S proteasome can be a drug target, where the administration of a low-dose proteasome inhibitor, such as bortezomib, significantly reduces the frequency of abdominal aortic aneurysms by decreasing the local inflammatory response^39^. Moreover, Li et al.^40^ also in mice model showed that the proteasome inhibitor bortezomib can partially attenuate abdominal aortic aneurysm model formation by modulating the infiltration of T lymphocytes through the regulation of ICAM-1 mRNA expression, which is controlled by the activation of the NF-κB signaling pathway^40^. The direct inhibition of the canonical NF-κB activation pathway leads to unwanted side effects due to the involvement of this pathway in many physiological processes as well.

In this context, the results of our studies on the activity of 20S ChT-L proteasomes can serve as inspiration for conducting research using proteasome inhibitors in the intracranial aneurysm model as well. Especially since there is currently no pharmacotherapy available that could reduce the number of necessary invasive procedures aimed at securing the aneurysm from rupturing^35^. The obtained results may serve as a basis for further research, especially in the context of proteasome inhibitor application. However, due to the small sample size, to confirm their significance for the entire population, it is necessary to continue the study in a larger research group.

### Study limitations

The study limitation could be the number of cases included to the study (n = 35, with 25 patients in the UIA group and 10 patients in the control group). The exploration of 20S proteasome ChT-L activity is considered “trailblazing” in patients with UIA. Consequently, we did not conduct a priori (prospective) power analysis and sample size calculations. However, it is usually done in big cooperative studies including cohorts.

The power analysis based on our results indicated a power of 0.65. Sample size calculations for a power of 0.8 revealed a recommended sample size of 19 for both the study and control groups. It is important to note that our results should be interpreted in the context of the study's limitations: the small number of patients, which may introduce a risk of selection bias. Moreover, that only CSF samples with a red blood cell count of 0/μl were used for further analysis, what significantly limited the number of patients included in the study.

## Conclusion

**Figure 5:**
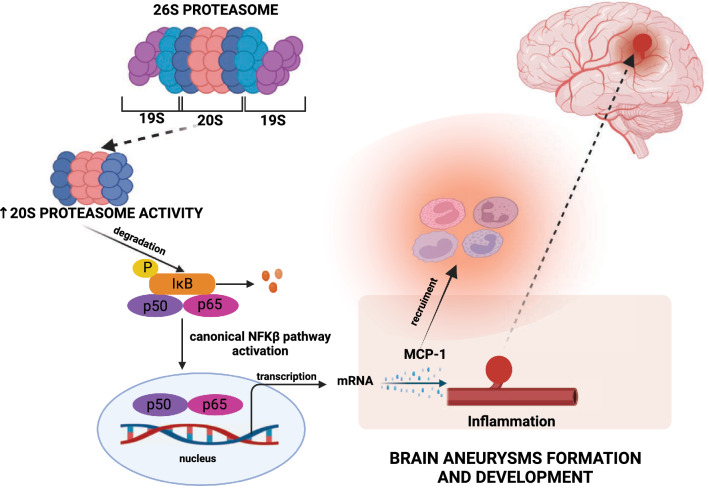
Suggested role of increased 20S proteasome activity in the formation and development of brain aneurysms. Figure was created with BioRender.com.

The results obtained by us may suggest that increased activity of 20S ChT-L proteasomes in patients with UIA modulates the inflammatory state in cerebral arteries through the activation of the canonical NF-κB pathway involving the NF-κB p50/p65 heterodimer. Furthermore, in patients with UIA, non-canonical NF-κB signaling, represented by the sequestered excess precursor subunit NF-κB2 (p100), may enhance the canonical activity of NF-κB (p65). These mutual interactions between the canonical and non-canonical NF-κB pathways may be involved in maintaining the inflammatory state in patients with UIA through the chemokine MCP-1, which is considered crucial in the formation and development of brain aneurysms (summarized in Fig. 5). Due to the promising results of our research on the activity of 20S ChT-L proteasomes, further research on the potential use of proteasome inhibitors as a therapy for patients with UIA should be considered. This would allow us to answer the question of whether 20S ChT-L proteasome could be a therapeutic target in the prevention or treatment of intracranial aneurysms.

## Material and methods

### Subjects

The research adhered to the guidelines outlined in the Declaration of Helsinki, and the protocol received approval from the Bioethics Human Research Committee at the Medical University of Bialystok (Permission No. APK.002.9.2021). Prior to their involvement in the study, all participants provided informed consent for their inclusion.

The study comprised a group of 25 individuals, consisting of 20 females and 5 males, who had unruptured intracranial aneurysms (UIA). The participants had an average age of 56 (range 30–70) years. All patients underwent craniotomy and direct surgical clipping to address their unruptured intracranial aneurysms. The surgical procedures were conducted at the Department of Neurosurgery located within the Clinical Hospital of the Medical University of Bialystok.

**Figure 6:**
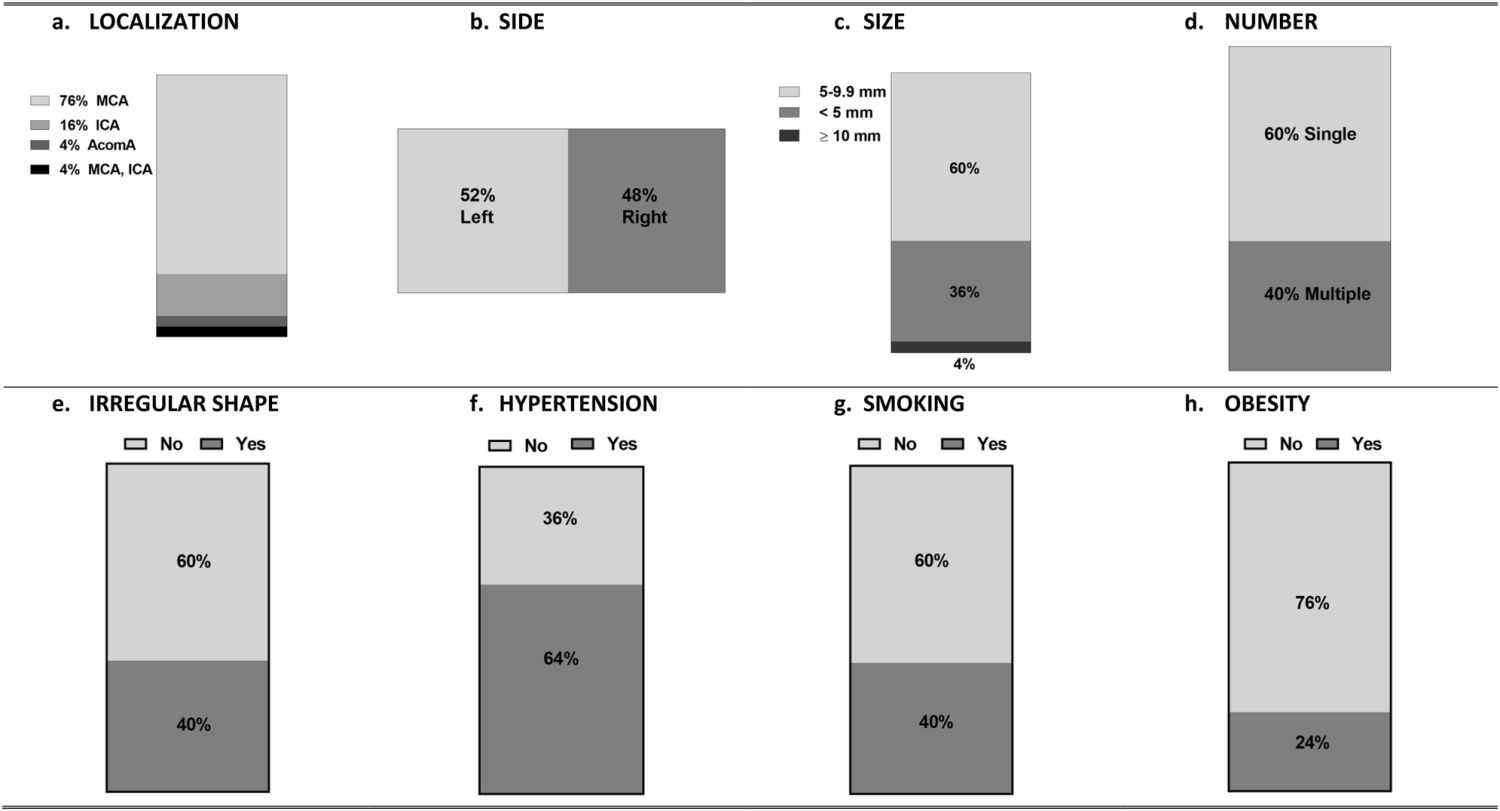
(**a–h**) The localization, side, size, number, irregular shape of brain aneurysms, and also risks factors of intracranial aneurysms development (hypertension, smoking, obesity) in the UIA patients group. Legend for Fig. 6 a-h.: MCA: middle cerebral artery, ICA: internal carotid artery, AcomA: anterior communicating artery. The aneurysm’s size is present for the biggest one. We identified the patient as a smoker if they smoked more than 20 cigarettes daily for a period of 12 months or longer. Individuals with a body mass index (BMI) exceeding 30.0 (kg/m2) were classified as obese.

In each patient, the considered aneurysms remained asymptomatic, discreetly positioning themselves at the forefront of the Willis circle. Validation of the UIA was achieved through computed tomography angiography for 14 patients and magnetic resonance angiography imaging for 11 patients. For a subset of eight individuals, an accurate delineation of the aneurysm's spatial coordinates, size, and morphology required confirmation via contrast digital angiography. The decision on whether to intervene hinged upon a meticulous evaluation of the delicate balance between the potential rupture of the aneurysm and the inherent risks associated with surgical procedures. This selection also takes into account the aneurysm's geometric characteristics, including dimensions surpassing 5 mm and/or assuming an irregular configuration/shape, indicative of an elevated susceptibility to rupture^41^, the subjective patient preferences, and also age, especially considering the lifelong risk associated with stent-graft in young individuals, if applied endovascular technique. The Fig. 6a–h depicts the localization, geometric characteristics, and risk factors associated with intracranial aneurysm development in the group of UIA patients (Fig. 6a–h). For more comprehensive details regarding the study please refer to the publication of Kamińska et al.^42^.

The control group included 10 individuals, comprising of 7 females and 3 males, who had an average age of 57 (range 25–70) years. These participants experienced trigeminal neuralgia due to an anatomical conflict between the trigeminal nerve and a cerebellar artery. Every patient within this group met the criteria for posterior fossa craniotomy and microvascular decompression. The procedure included exposing the conflict at the cerebro-pontine angle, detaching the compressing artery from the trigeminal nerve, repositioning the artery, and ultimately securing it to the cerebellar tentorium with an adhesive material (Tachosil®, Takeda, Linz, Austria).

Exclusion criteria were stringent for both groups, encompassing the presence of neurodegenerative conditions such as multiple sclerosis, a history of neuro-infections or brain tumor, recent surgery or major trauma in the preceding months, as well as the administration of antibiotics, anti-inflammatory drugs, or corticosteroids within the past months.

### Sample collection and storage

Blood samples were collected using 5.5 mL tubes without anticoagulant (S-Monovette, SARSTEDT) and 2.7 mL EDTA-K3 tubes (S-Monovette, SARSTEDT). Within 30 min of venipuncture, the blood was centrifuged for 20 min at 1000 × g to separate the serum and plasma. The obtained serum and plasma were subsequently stored at − 75 °C until analysis, the experiments were conducted just after thawing.

In both groups of patients, the cerebrospinal fluid was extracted from the subarachnoid space of the brain during craniotomy. Standard surgical procedures were employed, conducted under general anesthesia, with the patient's head secured in a three-pin Mayfield headholder. The process began with a skin incision, followed by the elevation of the bone flap and incision of the dura mater, facilitating the visualization of the arachnoid membrane and subarachnoid space. Using an operating microscope, the subarachnoid space was meticulously opened, and cerebrospinal fluid was aspirated using a single-use, sterile syringe and a soft venous catheter. Special precautions were taken to safeguard the cerebrospinal fluid against potential contamination from blood or the warm saline solution used for irrigation. To ensure the utmost purity, these measures were diligently executed at the beginning of each procedure, preempting any potential occurrence of bleeding. All the mentioned steps were carried out proactively at the beginning of each procedure to prevent any occurrence of bleeding. The cerebrospinal fluid samples underwent centrifugation for 20 min at 1000 × g. Following centrifugation, the resulting cerebrospinal fluid supernatant was divided into smaller aliquots and stored at a temperature of − 75 °C until further analysis.

### The plasma 20S proteasome chymotrypsin-like (ChT-L) activity evaluation

The chymotrypsin-like activity (ChT-L) of plasma 20S proteasome was evaluated by employing the fluorogenic peptide substrate Suc-Leu-Leu-Val-Tyr-AMC, along with a sodium dodecyl sulfate (SDS) selective enzyme activator, following the methodology described by Ma et al.^43,44^ and Tylicka et al.^2^. The addition of sodium dodecyl sulfate (SDS) was employed to activate the 20S proteasome in plasma due to its limited ability to efficiently degrade peptides or proteins that are not extensively denatured. The presence of SDS during activation aids in the penetration of the protein substrate into the proteasome channel. Plasma samples were activated by adding 5μL of 10% SDS and allowing it to incubate at room temperature for 15 min. The plasma activation phase involved using SDS at a concentration of 1%. Next, 10 μL of the samples were transferred to reaction wells containing 30 μL of an assay buffer (consisting of 0.05% SDS in 100 mM Tris/HCl, pH = 7.5) and 10 μL of the fluorogenic peptide AMC substrate. This resulted in a total volume of 50 μL for the reaction mixture, with an SDS concentration of 0.03% (the concentration required for optimal activation of the 20S proteasome). The chymotrypsin-like activity of the proteasome 20S cleaves the Suc-Leu-Leu-Val-Tyr-AMC substrate, resulting in the liberation of free AMC (7-amino-4-methylcoumarin). The quantity of liberated AMC was measured using a fluorescence microplate reader (FLUOstar OPTIMA, BMG Labtech, Germany). The reaction mixture was subjected to incubation at a temperature of 37 °C within a fluorescence microplate reader. During a 60-min incubation period, fluorescence measurements were taken at 3-min intervals. The fluorescence was monitored using an excitation wavelength of 355 nm and an emission wavelength of 460 nm. The rate of AMC release from the substrate per minute was quantified as one unit of the ChT-L activity of the 20S proteasome, denoted as pmol/min = U (units). The specific activity was determined by expressing the unit per milligram (U/mg) of total protein. The concentration of total protein in plasma samples was measured using the Bradford method, employing the Bio-Rad assay reagent with bovine serum albumin as the standard. The assays were conducted in triplicate. To confirm the specificity of the assay, plasma samples were pre-incubated with the selective proteasome inhibitor epoxomicin (1.0 μMol/L) for 15 min prior to the addition of the substrate, as per our earlier research findings^7^. In our previous study, we conducted measurements of plasma 20S proteasome activity both with and without the addition of SDS to verify if its inclusion resulted in a genuine increase in proteasome catalytic activity^45^.

### Precursor NF-κB1 (p105) and NF-κB2 (p100) proteins concentration evaluation

The concentrations of NF-κB1 (p105) and NF-κB2 (p100) were determined utilizing ELISA kits that employ the sandwich enzyme-linked immunosorbent assay method. The serum samples were subjected to analysis without undergoing any dilution beforehand. The experiments were carried out following the guidelines provided by the manufacturer. The concentrations of NF-κB1 (p105) and NF-κB2 (p100) were determined using human NFKB1 and NFKB2 kits (catalog numbers: orb563366 and orb563844, respectively) sourced from Biorbyt Ltd. in Cambridge, England. Both kits have a detection range of 0.0–20 ng/mL for NFKB1 and NFKB2, respectively. These assays demonstrate exceptional sensitivity and specificity in detecting NFKB1 and NFKB2, with no significant cross-reactivity or interference observed between NFKB1, NFKB2, and related analogs.

### Serum NF-κB p65 and cerebrospinal fluid MCP-1 concentration evaluation

The methodology of serum NF-κB p65 and cerebrospinal fluid MCP-1 concentration evaluation has been described in our previous publications^23,42^.

### Statistical analysis

The obtained results were analyzed with the use of the STATISTICA 13.0 PL software (StatSoft Inc., Tulsa, USA) and GraphPad Prism 5.0 (GraphPad Software, San Diego, USA). Due to the data not adhering to a normal distribution (confirmed by the X2-test), nonparametric statistical analyses were employed. The Mann–Whitney test was utilized to compare two independent samples, while Spearman's rank correlation was employed to obtain correlation coefficients. Values for continuous variables are presented as median with 25th and 75th percentiles (Q1 and Q3 quartiles). To assess the discriminative ability of plasma proteasome 20S ChT-L activity and serum NF-κB2 (p100) evaluation in differentiating between the UIA group and individuals without vascular lesions in the brain, a Receiver Operator Characteristic (ROC) curve was constructed. The Youden index, which combines sensitivity and specificity, revealed an optimal cut-off point between these two factors for the parameters under investigation. Statistical significance was determined at a significance level of < 0.05 for two-tailed p-values. The sample size was calculated with an independent two-sided t-test by choosing a 95% confidence level, a margin of error of ± 5%, power analysis of 80% (0.8). To the sample size calculation we used Sample Size Calculator ClinCalc.com from https://clincalc.com/stats/samplesize.aspx.

## Data Availability

The datasets generated and/or analyzed during the current study are not publicly available, but are available from the corresponding author (J.K.) on a request.

## Abbreviations

ACC: Diagnostic accuracy
AUC: Area under the ROC curve
ChT-L: Chymotrypsin-like
Cut-off: (Cut-off based on the highest Youden index)
IA: Intracranial aneurysms
MCP-1: Monocyte chemoattractant protein-1
NF-κB: Nuclear factor kappa-light-chain-enhancer of activated B cells
NPV: Negative predictive value
PPV: Positive predictive value
SDS: Sodium dodecyl sulfate
Se: Diagnostic sensitivity
SE: Standard error
Sp: Diagnostic specificity
UIA: Unruptured intracranial aneurysm
